# DSNetax: a deep learning species annotation method based on a deep-shallow parallel framework

**DOI:** 10.1093/bib/bbae157

**Published:** 2024-04-09

**Authors:** Hongyuan Zhao, Suyi Zhang, Hui Qin, Xiaogang Liu, Dongna Ma, Xiao Han, Jian Mao, Shuangping Liu

**Affiliations:** School of Artificial Intelligence and Computer Science, Jiangnan university, Wuxi, Jiangsu 214122, China; National Engineering Research Center of Cereal Fermentation and Food Biomanufacturing, State Key Laboratory of Food Science and Technology, School of Food Science and Technology, Jiangnan University, Wuxi, Jiangsu 214122, China; Luzhou Laojiao Group Co. Ltd, Luzhou 646000, China; Luzhou Laojiao Group Co. Ltd, Luzhou 646000, China; Luzhou Laojiao Group Co. Ltd, Luzhou 646000, China; National Engineering Research Center of Cereal Fermentation and Food Biomanufacturing, State Key Laboratory of Food Science and Technology, School of Food Science and Technology, Jiangnan University, Wuxi, Jiangsu 214122, China; National Engineering Research Center of Cereal Fermentation and Food Biomanufacturing, State Key Laboratory of Food Science and Technology, School of Food Science and Technology, Jiangnan University, Wuxi, Jiangsu 214122, China; Shaoxing Key Laboratory of Traditional Fermentation Food and Human Health, Jiangnan University (Shaoxing) Industrial Technology Research Institute, Shaoxing, Zhejiang 312000, China; National Engineering Research Center of Cereal Fermentation and Food Biomanufacturing, State Key Laboratory of Food Science and Technology, School of Food Science and Technology, Jiangnan University, Wuxi, Jiangsu 214122, China; Shaoxing Key Laboratory of Traditional Fermentation Food and Human Health, Jiangnan University (Shaoxing) Industrial Technology Research Institute, Shaoxing, Zhejiang 312000, China; School of Artificial Intelligence and Computer Science, Jiangnan university, Wuxi, Jiangsu 214122, China; National Engineering Research Center of Cereal Fermentation and Food Biomanufacturing, State Key Laboratory of Food Science and Technology, School of Food Science and Technology, Jiangnan University, Wuxi, Jiangsu 214122, China; Shaoxing Key Laboratory of Traditional Fermentation Food and Human Health, Jiangnan University (Shaoxing) Industrial Technology Research Institute, Shaoxing, Zhejiang 312000, China

**Keywords:** microbial species annotation, bioinformatics, natural language processing, deep learning, DNA sequence classification

## Abstract

Microbial community analysis is an important field to study the composition and function of microbial communities. Microbial species annotation is crucial to revealing microorganisms’ complex ecological functions in environmental, ecological and host interactions. Currently, widely used methods can suffer from issues such as inaccurate species-level annotations and time and memory constraints, and as sequencing technology advances and sequencing costs decline, microbial species annotation methods with higher quality classification effectiveness become critical. Therefore, we processed 16S rRNA gene sequences into k-mers sets and then used a trained DNABERT model to generate word vectors. We also design a parallel network structure consisting of deep and shallow modules to extract the semantic and detailed features of 16S rRNA gene sequences. Our method can accurately and rapidly classify bacterial sequences at the SILVA database’s genus and species level. The database is characterized by long sequence length (1500 base pairs), multiple sequences (428,748 reads) and high similarity. The results show that our method has better performance. The technique is nearly 20% more accurate at the species level than the currently popular naive Bayes-dominated QIIME 2 annotation method, and the top-5 results at the species level differ from BLAST methods by <2%. In summary, our approach combines a multi-module deep learning approach that overcomes the limitations of existing methods, providing an efficient and accurate solution for microbial species labeling and more reliable data support for microbiology research and application.

## INTRODUCTION

Microorganisms hold a crucial ecological niche within natural ecosystems, exerting an indispensable influence with profound ramifications for terrestrial ecosystems, human well-being and diverse industrial applications on Earth [1]. In the field of microbiology, bacteria are of undeniable importance. They are a remarkable group known for their ancient lineages, unparalleled diversity and widespread presence [2]. Nevertheless, this presents a complex challenge for the precise taxonomic classification of individual bacteria. Traditional approaches to microbial classification have relied on morphological and physiological criteria. However, this methodology has limitations, including subjectivity introduced by the observer’s judgment and experience and the inherent variability in microbial morphological and physiological characteristics. Furthermore, classification is limited to the genus and species level because traditional methods largely ignore the abundant genetic information and evolutionary relationships that underlie microorganisms.

Modern microbial species annotation methods have evolved to overcome these limitations, embracing cutting-edge methodologies rooted in molecular biology and genomics. Since the introduction of sequencing in 1975 [3], obtaining nucleotide sequences from microorganisms has become extremely easy. These sequences can be systematically compared to established sequences within databases, facilitating the precise classification of microbial species at the individual level. However, we must recognize that the time complexity of such an approach depends on several factors, including the size of the reference database, the amount of data requiring taxonomic annotation and the technical limitations of the comparison tool used.

The continuous development of sequencing technology allows us to obtain nucleotide and protein sequences. Significantly higher throughput and accuracy show that this area has made substantial progress. It is worth noting that the sequence length has changed considerably. In the past, it was only possible to obtain sequences of tens or hundreds of bases, but now it is possible to get sequences of >1000 bases. Consider the bacterial 16S rRNA gene, traditionally divided into 9 variable regions (V1-V9) and 10 conserved regions (C1-C10). Before the advent of the third-generation sequencing technology, we could only obtain short areas such as V1-V3, V3-V5 and V4. However, with the introduction of third-generation sequencing technology, researchers can now capture the 16S rRNA gene sequence containing about 1500 bases [4]. The development of this technology not only improves the depth and comprehensiveness of sequencing and can provide researchers with more comprehensive and precise information about microbial communities.

In addition, the exponential growth in the quantity of sequence data poses a significant challenge for species annotation. The rapid accumulation of data adds complexity, necessitating more precise and efficient classification methods to handle these large-scale sequence data. Thus, it necessitates an ongoing endeavor to refine and develop novel algorithms and technologies to cope with the overwhelming amount of data generated by modern sequencing techniques [5]. With the constant progress of genome sequencing technology, we could obtain more widespread species genome sequence information. Such rich data resources contribute to a more comprehensive understanding of the complex network of microbial diversity and the different functional characteristics that microbes possess. These developments highlight the vital role played by contemporary genomics and bioinformatics in uncovering the mysteries of microbial life on Earth.

Contemporary microbial species annotation methods can be divided into three categories depending on the sequence extracted: marker gene-based methods, whole genome sequence-based methods and methods utilizing protein sequences. Furthermore, these methods can be classified according to their algorithms, which typically fall into three categories: similarity comparison-based, based on traditional machine learning and deep learning-based methods. Among these, the most prevalent approach is the algorithm based on similarity comparison. This method achieves high levels of classification accuracy by evaluating the similarity between known and unidentified sequences. However, it must be emphasized that achieving this excellent accuracy requires a significant time investment and heavily relies on accurate and well-developed dataset support.

Beyond conventional similarity comparison methods, exploring novel classification techniques, including traditional machine learning and deep learning, can provide additional avenues and tools for the more precise annotation and interpretation of diverse bacteria within microbial samples [6–8]. Sustained research and technological innovations are poised to propel the advancement of microbial taxonomy. In turn, it will contribute to a deeper understanding of microbes’ critical role in complex natural and ecosystem networks. These advances help fully exploit microorganisms’ potential for a wide range of applications and play an essential role in promoting scientific research and practical applications in related fields.

However, traditional machine learning methods for microbial classification have several challenges, one of the main issues being the need to manually select different features or use different algorithms to generate feature datasets. These features may cover gc content, species abundance information and k-mer probabilities based on naive Bayes. However, feature selection and dataset generation usually require considerable time and effort and sometimes even manual construction. As our knowledge of microbial diversity continues to expand, more and more species are being discovered, making it more challenging to distinguish microbes at the species level. In some cases, traditional machine learning methods require more feature data to achieve the necessary level of analysis. This may lead to the processing of high-dimensional data with many samples, increasing the computational complexity. This highlights the urgent need to develop more efficient and accurate annotation techniques to handle increasing microbial sequence data.

Facing these challenges, current research directions include exploring new feature extraction methods, optimizing algorithms to reduce computational costs and introducing advanced techniques such as deep learning to improve the effect of microbial classification. Such innovative efforts are expected to increase the automation of microbial classification and reduce the burden of human intervention to cope more effectively with the growing and complex microbial data.

In addition to traditional methods for constructing feature datasets, new feature construction techniques have emerged. One such approach involves sequential encoding, where nucleotides are replaced with distinct numerical values, such as [0.25, 0.5, 0.75, 1] and other characters like ‘N’ can be substituted with 0. Another widely adopted method is one-hot encoding, which transforms nucleotide bases into four-dimensional arrays [9]. Recent advances have introduced another popular method of splitting 16S rRNA gene sequences into k-mers sequences and then treating that k-mers sequence as a linguistic text, applying techniques from natural language processing (NLP) [10]. This innovative feature construction approach is expected to improve the accuracy and efficiency of microbial taxonomy by leveraging contextual information embedded in k-mers sequences [11].

Deep learning techniques also have made significant progress in species annotation methods in recent years, mainly because of their ability to model intricate relationships within data. An illustrative example of a deep learning-based method is DeepMicrobes [12], a classification approach tailored to gut microbial data. DeepMicrobes utilizes a collection of k-mers, with k set to 12, for making classifications. Another notable method is BERTax [13]. They offer the flexibility to either utilize pre-trained models for annotation against specific microbial datasets or to conduct model training directly using the designed database. However, BERTax adopts a k-value of 3 and demonstrates superior classification results compared to DeepMicrobes. They illustrate the continued refinement and optimization of deep learning methods in microbial species annotation, highlighting the potential of deep learning techniques to improve the accuracy and effectiveness of these methods.

This paper introduces DSNetax, a novel approach for species-level classification of 16S rRNA gene sequences. To convert the 16S rRNA gene sequence into a numeric vector that a computer can recognize, we use DNABERT^15^ for the conversion, which is based on the Bidirectional Encoder Representation from Transformers (BERT) architecture, a state-of-the-art NLP architecture. After converting 16S rRNA gene sequences into ‘linguistic texts’ [14], DSNetax performs microbial species classification based on this unique biolinguistic perspective. This process unfolds through carefully designed deep and shallow modules parallel network classification models. The annotation results of bacteria within communities obtained using DSNetax show high accuracy at both genus and species levels.

## MATERIALS AND METHODS

**Figure 1:**
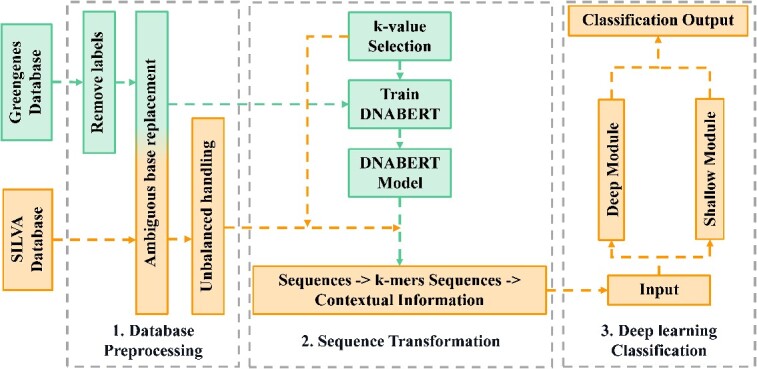
Data flow diagram. We train two different deep learning models using two different datasets, where the Greengenes database is used to train the DNABERT model and the SILVA database is used to train our classification model.

**Figure 2:**
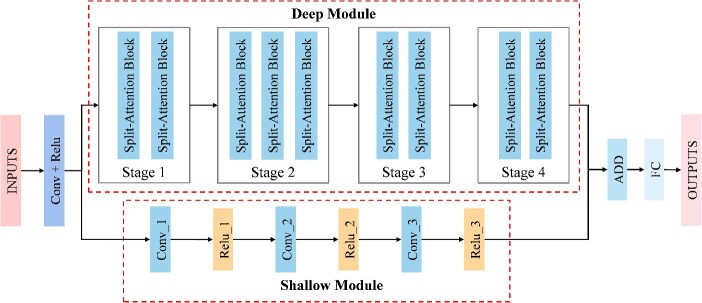
DSNetax model structure diagram. DSNetax is a parallel structure of deep and shallow modules. The deep module consists of four stages, each containing, in turn, 2, 3, 2 and 2 Split-Attention blocks. The shallow module has three successive convolution operation (Conv) and Rectified Linear Unit (ReLU).

DSNetax is based on the ResNeSt [15] framework, which significantly improves performance compared to previous models without substantially increasing the number of parameters. Many downstream applications, such as object detection and image segmentation, have achieved excellent results. We combine our method, DSNetax, with data processed by sequence language models, taking into account both data processing and methodology (Figure 1). The overall framework diagram of DSNetax is shown in Figure 2, which skillfully integrates deep semantic insights while preserving complex low-level details.

### Training ‘16S rRNA Gene’ sequence language models

In this study, we adopt DNABERT [16] as our preferred model for training sequence languages. DNABERT is a derivation of the recent BERT architecture that removes the next sentence prediction (NSP) task while retaining the masked language Modeling (MLM) task. The MLM task involves randomly masking tokens (words or sentence fragments) in a sentence and then predicting these masked tokens. The NSP task allows us to understand the relationship between two sentences. While this is valuable for downstream functions that rely on sentence relationships, many studies have confirmed that for non-two-sentence forms of tasks, deleting NSP tasks will improve the performance of downstream tasks [17].

DNABERT imposes specific prerequisites for processing sequences, requiring them to be treated as k-mers sets. Additionally, to facilitate training, five distinct tokens are indispensable: the classification token [CLS], the mask token [MASK], the padding token [PAD], the unknown token]UNK] and the separator token [SEP]. [CLS] is used to indicate the ‘meaning’ of the entire sentence. [MASK] masks words that need to be predicted in the MLM. The [PAD] represents filling the sentence length to a specific value. The [UNK] means the unknown token in the sentence. Moreover, the separator marker [SEP] separates two sentences in the NSP task.

### Data

#### Bacterial 16S rRNA gene sequence language data

To convert 16S rRNA gene sequences into numerical vectors suitable for deep learning classification models, we use the Greengenes database to train the sequence language model. Although this database may have less content and a slower update frequency than alternatives, it boasts superior data quality. More significantly, it is advantageous in its broad suitability for training embedding models, as outlined in prior research [18].

#### Bacterial 16S rRNA gene sequence classification data

For the bacterial data sets, several prominent databases are available, including the Ribosomal Database Project (RDP) [19], Greengenes [20], SILVA [21] and EzBioCloud [22], among others. After considering factors such as update frequency and sequence quantity, we select version 138.1 of the bacterial 16S rRNA gene sequence database from the SILVA. This version also includes the corresponding microbial annotations. The sequence database follows the standard FASTA format, where each data entry consists of a Feature ID and the associated 16S rRNA sequence. The annotation information database consists of two columns. The first column corresponds to the Feature ID found in the sequence database, while the second column provides taxonomic information in the following format, for example d__Bacteria; p__Actinobacteriota; c__Actinobacteria; o__Streptomycetales; f__Streptomycetaceae; g__Streptomyces; s__Streptomyces_phaeochromogenes.

### Bacterial 16S rRNA gene sequence database pre-processing

In the database we obtain, two critical processing aspects were necessary. First, there is a need to handle ambiguous bases. The SILVA database contains ambiguous bases and nucleotide bases. These ambiguous bases are usually represented by a symbol, representing multiple nucleotide bases. An excessive amount of ambiguous bases in a sequence, compared to a complete nucleotide sequence, can lead to a loss of partial information. This situation can lead to smooth fluctuations in feature data within a specific range. When converting a sequence into k-mers and selecting an enormous value of k, the transformation into a numeric vector matrix may result in extended segments of unchanged vector values. It is because, in the language model for 16S rRNA gene sequences, a k-mer that contains such a base is usually labeled as a [UNK] tag.

Second, handling unbalanced databases was essential. The SILVA database consists mainly of data provided by the laboratory and shared data from other databases. The sequence counts may vary significantly among different species, and some species may have only one sequence sample. When dividing the database into training and test sets, such imbalance may introduce bias and potentially result in insufficient learning for certain samples. To address these issues, we implemented the following data processing steps:

(i) The ambiguous bases of species with 11 or more sequences are randomly replaced with nucleotide bases.
(ii) For species with a small number of sequences (less than or equal to 10), we replicated the sequences multiple times based on the number of sequences for each species (Table 1). We then randomly replaced the ambiguous bases in these replicated sequences.

**Table 1:** The number of repetitions of species with fewer sequences

Number of sequences contained in the remaining species	Number of repetitions
1,2	12
3,4,5	4
6, 7, 8, 9, 10	2

The results of these processing steps are summarized in Table 2, where the number of sequences in the SILVA database used for classification model training is more than doubled. These measures are essential to alleviate the problem of ambiguous bases and unbalanced distribution in the database.

**Table 2:** Comparison of SILVA and Greengenes databases before and after preprocessing

	SILVA 138.1	Greengenes
Number of species	45 738	/
Number of sequences before processing	428 748	203 452
Number of sequences after processing	998 503	203 452

### Bacterial 16S rRNA gene sequence database post-processing

#### Database split processing

The next step in preparing the database for training deep learning classification models involves the following steps. The database is divided into two subsets: a 90% training set and a 10% test set. The dataset is split using the stratify parameter to ensure all species can be learned and classified. Stratified splitting ensures that species distribution remains consistent between the training and test sets. It is particularly crucial when there is data imbalance, as it helps prevent certain species from being excluded from the training or testing data.

#### 16S rRNA gene sequence conversion processing

After completing the preprocessing of the database, the next step is to transform the sequence data into numerical vectors suitable for computer recognition. The one-hot encoding method, which has been widely used with significant success in applying deep learning to NLP tasks, has two main drawbacks. Firstly, the curse of dimensionality becomes prominent, especially when dealing with large vocabularies. Secondly, this representation struggles to capture word similarities, hindering the model’s ability to understand contextual variations. Therefore, word embedding technology emerges as a second representation method.

The core idea of word embeddings is to represent each word as a plain vector, which, unlike one-hot vectors, contains actual values instead of binary ones. This representation embeds words into the mathematical space such that similar words are also closer in the vector space. While processing the sequence, we convert it into a set of k-mers and treat this k-mers set as a biolinguistic text. These k-mers are then transformed into word vectors using a biological language model, implementing a word embedding process in natural language [14]. Notably, this transformation approach will better capture contextual information in 16S rRNA sequences and provide more affluent and more accurate feature representations for subsequent applications of deep learning models.

### Microbial species annotation model structure

In our approach to annotating bacterial 16S rRNA gene sequences, we have chosen the ResNet [23] model and its variant, the ResNeSt [15] model, as the foundational models. These methods offer a distinct advantage over traditional methods that heavily rely on feature engineering. They can automatically learn relevant features directly from the data, making them well-suited for large datasets. In our DSNetax architecture, we have adopted the ResNeSt network structure, demonstrating superior performance to conventional ResNet in computer vision tasks, especially in image classification. This improved performance is achieved by incorporating a referential split-attention mechanism and multi-scale feature integration without significantly increasing the number of parameters.

The core architecture of DSNetax is a parallel structure of deep and shallow modules. The deep module consists of four stages, each containing, in turn, 2, 3, 2 and 2 Split-Attention blocks, a configuration designed to capture global features from input data efficiently. In contrast, the DSNetax shallow module is built on convolutional neural networks (CNNs). It consists of three successive convolution operation (Conv) and rectified linear unit. This arrangement is designed to facilitate smoother transitions between features in the data set, minimize loss of information and capture local features.

Crucially, the architecture combines the global semantic features extracted by the deep module and the local detail features captured by the shallow module. This integration aims to create a comprehensive overall feature fusion representation, which leverages the complete understanding provided by the global features while accommodating the nuances and fine-grained details captured by the local features. It is central to the success of DSNetax in effectively annotating bacterial 16S rRNA sequences.

## RESULTS AND DISCUSSION

**Figure 3:**
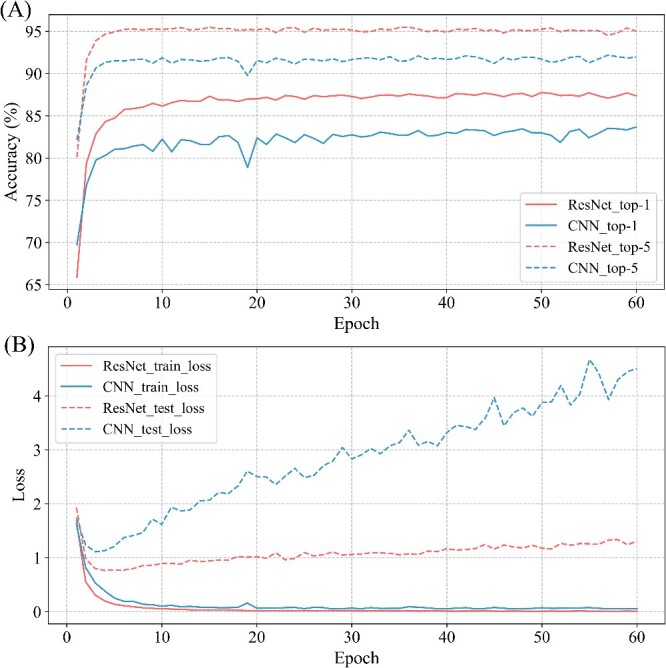
Accuracy and loss of ResNet (34) model and CNN model. (**A**): Accuracy of ResNet (34) model and CNN model. (**B**): Loss of ResNet (34) model and CNN model.

First of all, to verify that more complex deep learning models may bring better experimental results, we used the 3-layer CNN model and ResNet (34 layers) model to conduct a comparison experiment (Figure 3). At this time, we performed 3-mers processing on 16S rRNA sequences and converted them into word vectors by using the trained DNABERT model. As shown in Figure 3A, although the ResNet (34 layers) model maintained a lower state than the CNN model at the beginning of training, it quickly catches up with the CNN model and maintains a better state than CNN all the time. Therefore, complex models can have better learning results. For the ResNet (34 layers) model, the model tends to be stable when it reaches 30 rounds, but there is still a 1–2% improvement when it comes to 60 epochs of training. Consequently, we determined the number of subsequent model training to be 60 epochs.

Then, we find that the training set loss of both models decreases during the training iteration. However, the loss of test sets drops and then increases. Excessive sequence similarity in the data set may cause this behavior. In other words, simple networks are very prone to overfitting, while complex networks alleviate this situation [24]. Due to the potentially enhanced feature extraction capabilities of complex networks, the ResNet (34 layers) model exhibits a significantly smaller increase in test set loss compared to the CNN model. It shows that the ResNet model is more advantageous in dealing with similar databases (Figure 3B).

**Figure 4:**
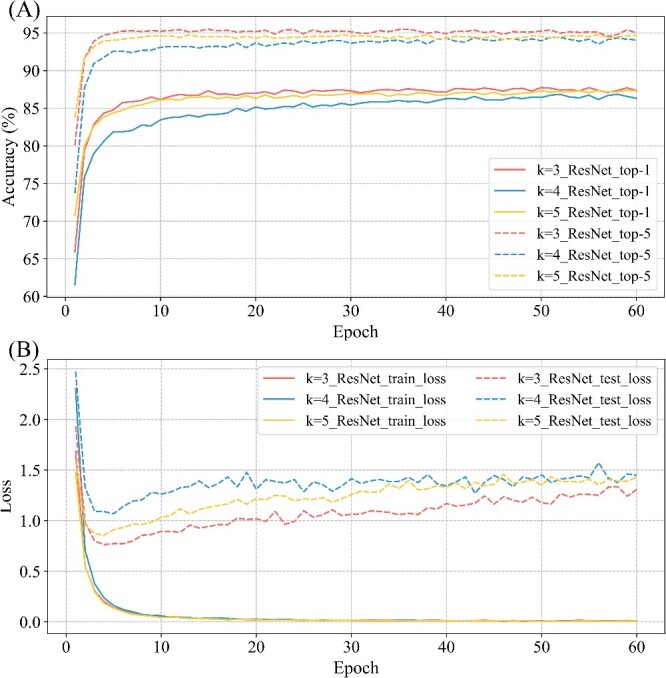
Comparison of results of ResNet (34) model with different k values. (**A**): Accuracy of ResNet (34) model with different k values. (**B**): Loss of ResNet (34) model with different k values.

We already know that complex models can lead to better classification. To find the optimal value of k that can improve the recognition accuracy of our model, we conduct experiments based on the ResNet (34 layers) model. Considering that a small value of k may not be sufficient to provide sufficient information, and a considerable value of k is too demanding on the configuration of the hardware, we chose additional k = 4 and 5 for testing [25]. The results are shown in Figure 4. When k = 3, the top-1 and top-5 results are higher than when k = 4 and 5. However, there is little difference in top-1 or top-5 accuracy for different k values. Secondly, no matter how much k is set, there is a significant difference in recognition accuracy between top-1 and top-5. This difference is about 10%, and we believe that distinguishing 16S rRNA sequences is a challenging task (Figure 4A).

In addition, the loss on the training set still decreases and then stabilizes, while the loss on the test set decreases and then increases. This observation is consistent with the results of previous experiments. However, we notice that the test set loss for k = 3 is always lower than the loss for k = 4 and k = 5 (Figure 4B). Therefore, k = 3 can lead to better classification results.

**Figure 5:**
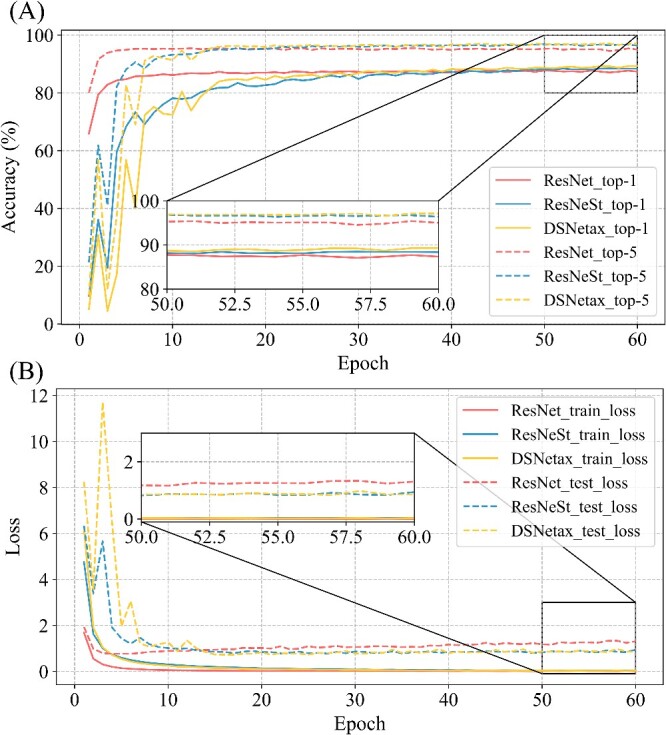
Comparison of ResNet, ResNeSt and DSNetax model results. (**A**): Accuracy of ResNet, ResNeSt and DSNetax. (**B**): Loss of ResNet, ResNeSt and DSNetax.

Although previous experiments show that complex models can lead to better recognition accuracy, the problem of overfitting and low accuracy still cannot be avoided. Therefore, we chose the more complex ResNeSt model for our experiments while also adopting our own DSNetax model, the results of which are shown in Figure 5. The top-1 and top-5 recognition accuracies of the three models are not significantly different, which indicates that the three models have essentially the same classification performance. However, DSNetax consistently outperforms the other two models after >30 rounds of training (Figure 5A).

In addition, the training set loss of all three models drops to zero, indicating that these models have effectively learned knowledge from the training data. However, the test set loss exhibits a different pattern: ResNet’s test set loss trend is consistent with the previous trend. In contrast, the ResNeSt and DSNetax models exhibit similar early fluctuating trends in test set loss, followed by a steady decline to 1 in the later period (Figure 5B). In other words, the more complex model effectively avoids the overfitting problem and achieves better recognition accuracy than the ResNet model. Our results highlight the competitive performance of DSNetax, consistently outperforming the other two models over 30 training rounds. It demonstrates the good performance of DSNetax for 16S rRNA gene datasets with high sequence similarity. It also highlights the importance of using complex network architectures when dealing with this data type. These findings suggest that the final results can be improved by increasing the model structure’s complexity to accommodate the dataset’s complexity and subtleties.

**Figure 6:**
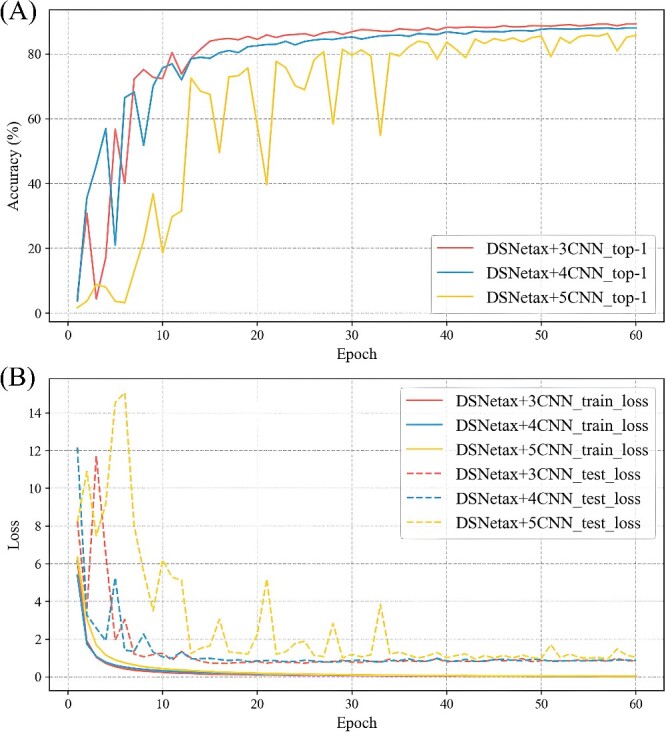
Accuracy and loss of DSNetax+3CNN, DSNetax+4CNN, DSNetax+5CNN. (**A**): Accuracy of DSNetax+3CNN, DSNetax+4CNN, DSNetax+5CNN. (**B**): Loss of DSNetax+3CNN, DSNetax+4CNN, DSNetax+5CNN.

Through the previous experimental results, we observe that the DSNetax model has achieved superior results in classification tasks, directly demonstrating our model structure’s good performance. To further explore the influence of different levels of shallow module on the classification effect, we conduct further experiments by replacing the 3-layer CNNS of DSNetax with 4-layer and 5-layer CNNS for comparison. For convenience of comparison, we name DSNetax with 3-layer CNN as DSNetax+3NN, and so on. The experimental results show that the recognition accuracy of DSNetax+3NN is always higher than that of the other two structures, which further verifies the excellence of our proposed model structure (Figure 6). In deep learning, increasing layers usually makes the network more capable of extracting abstract semantic features. However, our experiments prove that the 3-layer CNN adopted in DSNetax is already sufficient for the microbial classification task to effectively extract the critical detailed features. Further increasing the number of layers of CNN may lead to features that are more inclined to the semantic level and not conducive to capturing detailed features. This balance is achieved between global semantic features and local detail features, emphasizing the outstanding performance of the DSNetax model on the task.

**Figure 7:**
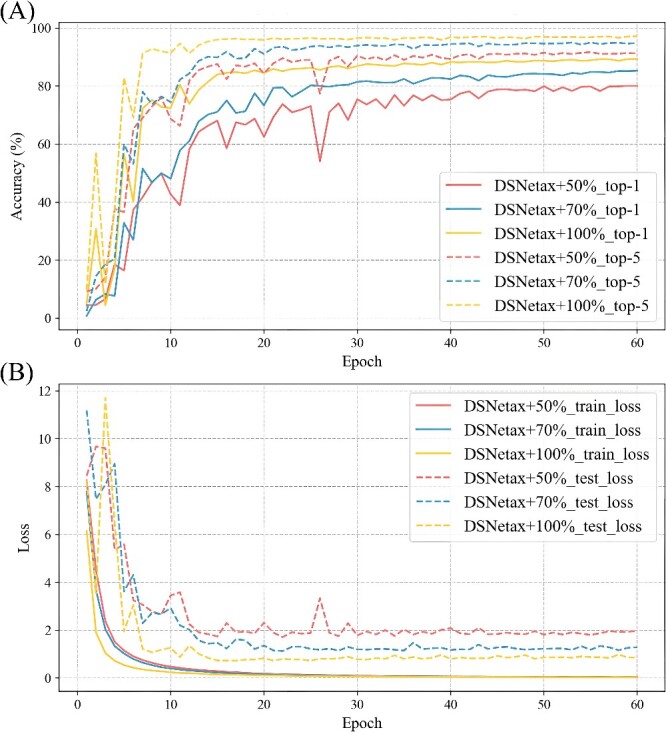
DSNetax accuracy and loss on 50%, 70%, 100% of the training data sets. (**A**) DSNetax accuracy on 50%, 70%, 100% of the training data sets. (**B**): DSNetax loss on 50%, 70%, 100% of the training data sets.

The advantage of DSNetax is its deep and shallow parallel module structure, and the drive of big data escorts DSNetax. Therefore, to explore the impact of data volume on DSNetax performance, we split the original training set according to the proportion of 50 and 70%, and the result is shown in Figure 7. We find that more training data leads to better recognition. This result indicates that performance can be improved further as the available data increases. The bacterial sequences may not differ much before and after the variation of variable region bases. Therefore, training the bacterial species annotation model requires more training data to ensure the model’s generalization.

**Figure 8:**
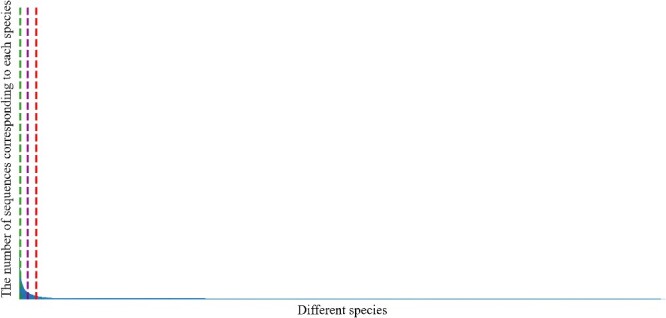
Long tail data presentation of the training set. The green, purple and red dashed lines respectively denote the segmentation positions at 500, 100 and 50.

However, our dataset also presents another feature: the long-tail distribution (Figure 8). The long-tail distribution refers to the scenario where the head categories have a larger number of samples, while, in contrast, categories in the tail have only collected a small number of samples. Figure 8 illustrates the distribution of data in the training set and the segmentation positions. The vertical axis represents the number of sequences for each species, while the horizontal axis represents species arranged in descending order based on the corresponding sequence counts. Most of the data collected in the bacterial 16S rRNA database is typical or has been studied many times. Due to the large number of species in the database we used, the dataset distribution shows a long tail and a steep head. Therefore, according to the long-tail distribution of the training set, we divide the test set into two parts, head and tail and test the model. We segmented header and tail data three times, and the experimental results are shown in Table 3. We choose 50, 100 and 500 as the cutting points (In Figure 8, the red, purple and green dashed lines represent split locations at 50, 100, and 500, respectively).

**Table 3:** Accuracy (%) of split head and tail data at different positions

	top-1	top-5
HEAD (≥ 50 reads)	78.67	96
HEAD (≥ 100 reads)	81.15	97.06
HEAD (≥ 500 reads)	86.83	98.8
TAIL (< 50 reads)	93.03	97.5
TAIL (< 100 reads)	91.48	97.14

In general, the head of long-tail data should have higher accuracy, but in our experiment, the accuracy of tail data is more elevated. Before splitting the dataset, ambiguous bases in high-abundance species (those with >11 sequences, including 11 sequences) are randomly replaced. But low-abundance species (<10 sequences, including 10 sequences), we duplicated sequences according to the number of sequences for each species and then randomly replaced ambiguous bases. However, the resulting sequence bases are mostly the same, leading to a higher sequence similarity. Additionally, some sequences do not contain ambiguous bases, and these sequence data are repeated in the samples, potentially causing an excessive similarity between the training and testing sets. As a result, samples from low-abundance species exhibit relatively high similarity, contributing to the model’s good classification performance during testing.

Our method has demonstrated decent annotation accuracy at the species level in the final comparison with existing popular methods using the divided SILVA dataset. However, at the genus level, it appears slightly less accurate than the microbial species annotation method integrated into Qiime 2 [26, 27]. Meanwhile, the BLAST method [28, 29] has demonstrated outstanding annotation outcomes at both the species and genus levels. Qiime 2’s method prioritizes annotation speed over accuracy, resulting in slightly lower accuracy than the BLAST method. This trade-off is often necessary in large-scale microbial annotation tasks, where computational efficiency is crucial. DSNetax has demonstrated the capability to achieve top-5 results at the species level that are broadly comparable to those of the BLAST method. This finding suggests that DSNetax represents a promising direction for future optimization work, particularly in scenarios where accuracy and speed are crucial (Table 4).

**Table 4:** Accuracy (%) of tools or methods on SILVA database

		Species	Genus	Time
DSNetax	top-1	89.37	96.26	0.76 h
	top-5	97.12	/	
Qiime 2	/	69.46	97.1	0.87 h
BLAST	/	98.87	99.98	73.83 h

**Figure 9:**
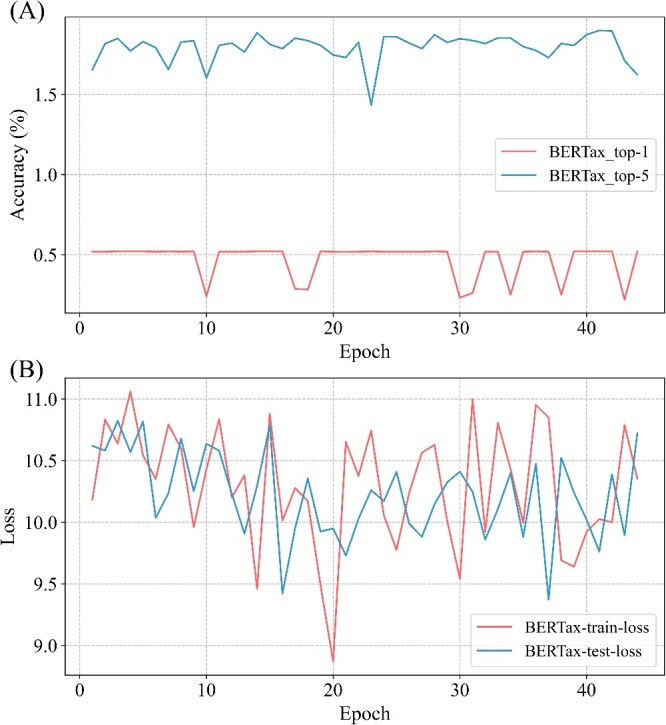
BERTax model results. (**A**): Accuracy of BERTax. (**B**): Loss of BERTax.

We compared the state-of-the-art deep learning method BERTax in our study. However, it has limitations, primarily when dealing with large datasets. These limitations arise from factors such as model constraints and dataset characteristics. For instance, BERTax has a restriction where the input is limited to 512 or fewer tokens. As the data lengths from third-generation sequencing exceed this limit, additional operations may be needed to handle longer sequences, potentially leading to information loss. Furthermore, the SILVA dataset comprises many highly similar sequences, posing challenges for DSNetax and similarly for methods like BERTax (Figure 9).

Overall, this comparison highlights the trade-offs and strengths of various annotation methods, offering valuable insights into the benefits and areas for enhancement in each approach. It also underscores the potential of DSNetax as a competitive method for microbial species annotation, particularly when striking a balance between accuracy and computational efficiency is essential.

Unlike the favorable results achieved with the SILVA database, our method does not show an advantage over the GreenGenes and RDP databases. This discrepancy indicates that the effectiveness of our process may be sensitive to the size and balance of the dataset. In cases where the dataset is small, extremely imbalanced and needs more training data, achieving the necessary level of training for a deep learning model can be challenging. Despite not outperforming other methods on these smaller databases, our method consumes less time. This indicates its potential as a time-efficient choice for annotation tasks. Moreover, even when applied to these challenging databases, our method maintained a certain level of accuracy. Such results hold practical value in real-world applications (Table 5).

**Table 5:** Accuracy (%) of tools or methods on Greengenes and RDP database

	Greengenes			RDP		
	Species	Genus	Time	Species	Genus	Time
DSNetax	72.65	92.17	1 min	85.49	96.11	3 min
Qiime 2	90.6	98.24	1 min	89.61	99.57	5 min
BLAST	91.6	99.03	53 min	94.36	99.63	33 min

## CONCLUSION

DSNetax builds a deep learning classification model based on a unique biological language perspective, aiming to balance annotation time and accuracy in microbial species annotation. Our method performs well on top-1 results and achieves almost species-level annotation results like BLAST on top-5. However, while the concept of 16S rRNA gene sequences as natural language is practical, there is still room for improvement and fine-tuning in practical applications. Future studies may focus on further improving the applicability of this method.

Second, we observed that larger deep classification models showed superior performance. However, they may need to steadily show their strength when dealing with small and imbalanced data sets. With the challenges of data explosion, especially in the era of big data, dealing with extensive data integration becomes an issue worthy of attention. Future research could explore strategies for efficiently handling large data sets, such as data sampling techniques, distributed computing, or model architectures designed to handle large amounts of data.

We mentioned that combining different feature datasets and deep learning classification model to create comprehensive approaches is promising. This hybrid approach is expected to provide better performance and flexibility in processing additional microbial data. Finally, using NLP structures to process the long data input of biological language sequences is an essential area of future research. This adaptation is necessary to ensure that NLP technology can effectively deal with the uniqueness of biological sequences.

DSNetax is a promising microbial species annotation approach that successfully addresses the need for a balance between annotation time and accuracy. It simplifies the annotation process, improves the species-level classification performance, and optimizes the utilization of computing resources. Our work provides valuable insights into the potential and challenges of using 16S rRNA gene sequences as NLP.

**Key Points**

- Combining the biological language model with a deep learning classification model that can extract deep semantic and shallow detail information has improved the method for microbial species annotation tasks.
- The top-5 results of the DSNetax model closely align with the BLAST method, indicating a promising direction for advancing microbial species annotation.
- DSNetax can balance speed and accuracy in annotating microbial species at the species level.

## Data Availability

The additional data and code related to this paper can be downloaded from https://github.com/ZhaoHY-zhy/DSNetax.

## Author Biographies

** Hongyuan Zhao** is a graduate student at the School of Artificial Intelligence and Computer Science at Jiangnan University, specializing in deep learning and bioinformatics research.

**Suyi Zhang** is a professor-level senior engineer at the School of Bioengineering, Sichuan University of Technology. His research interests are brewing microorganisms and their applications.

**Hui Qin** is deputy general manager of the brewing company of Luzhou Laojiao Group Co., LTD.

**Xiaogang Liu** is deputy general manager of Luzhou Laojiao Health Wine Company.

**Dongna Ma** is an assistant researcher at the School of Food Sciences, Jiangnan University, focusing on food microbial molecular ecology and bioinformatics.

**Xiao Han** is an experimentalist at the School of Food Sciences at Jiangnan University, specializing in food biotechnology and food engineering.

**Jian Mao** is a professor at the School of Food Science at Jiangnan University, specializing in brewing food and Marine special protection food.

**Shuangping Liu** is a professor at the School of Food Science and Technology, and Associate Professor at the School of Artificial Intelligence and Computer Science, Jiangnan University. His main research interests include molecular ecology of food microbiology, synthetic microbiome research and industrial technology development.
